# Electronics for Sensors

**DOI:** 10.3390/s21062226

**Published:** 2021-03-23

**Authors:** Giuseppe Ferri, Gianluca Barile, Alfiero Leoni

**Affiliations:** Department of Industrial and Information Engineering and Economics, University of L’Aquila, 67100 L’Aquila, Italy; gianluca.barile@univaq.it (G.B.); alfiero.leoni@univaq.it (A.L.)

Research on systems and circuits for interfacing sensors has always been, and will surely be, a highly prioritized, widespread, and lively topic. This is because each application that has usefulness in the real world inevitably needs to perceive and elaborate some kind of magnitudes coming from it. For this reason, however, advances in technology in many fields continuously pose new challenges in designing electronic interfaces. In particular, they have to satisfy parameters strictly related to the magnitude under evaluation, such as sensitivity and resolution, in addition to fulfilling new constraints coming from the particular macro-system into which the sensor interface is embedded, such as low power consumption and low cost. To make it even more challenging, with the first interfacing stage typically analog, it does not gain much benefit from the technology scaling in terms of chip area reduction and power consumption, whereas it has typically to deal with parasitic elements that are of the same magnitude as the sensing element. Therefore, the aim of this Special Issue has been to gather works that present new possible solutions regarding electronics for sensors, both related to the analog processing stage as well as including the digital processing section. Published manuscripts well embody the advances and the latest novel and emergent results on the Special Issue topic, showing best practices, implementations, and applications.

This Special Issue contains a selection of 16 papers covering photomultiplier tubes and silicon photomultipliers (PMTs and SiPMs) interfaces and applications [1,2,3], techniques for monitoring radiation levels [4,5], electronics for biomedical applications [6,7,8], design and applications of time-to-digital converters [9,10,11], interfaces for image sensors [12,13] and general-purpose theory and topologies for electronic interfaces [14,15,16]. 

In [1], Bosch et al. describe how it is possible to use together photomultiplier tubes and silicon photomultipliers in a high-pressured Xenon particle detection chamber as part of the Neutrino Experiment with a Xenon Time projection chamber (NEXT)–White experiment. In [2], Calò et al. address how it is possible to take full advantage of SiPMs intrinsic fast response time for characterizing and modeling the same SiPM when parasitic elements are associated with the interconnection between the sensor and the interface degrade its performances. In [3], Stornelli et al. propose a discrete-transistors level electronic interface for SiPMs. The interface is based on the novel active block called second-generation voltage conveyor (VCII) and has therefore the capability of working both in voltage and current mode taking advantage of both approaches. 

In [4], Jeon et al. propose a power-efficient interface for battery-powered dosimeters. The manuscript presents both the analog and the analog-to-digital conversion stages and proposes a sampling scheme that allows the analog to digital converter (ADC) to only operate near its peak value. In [5], Holovatyy et al. design and implement a microcontroller-based radiation monitoring system that is able to detect if excessive radiation exposure is experienced in order to prevent long-term damages. 

In [6], Castro et al. propose a novel bioreactor for biological tissue engineering that is able to remotely stimulate magnetoelectric scaffolds through a magnetic field, and therefore, via the scaffolds, provide a mechanical or electrical stimulus to the cells contained in the reactor. In [7], Zhan et al. propose a novel methodology for indoor gas analysis based on a one-dimensional convolutional neural network, capable of detecting harmful gas components by extracting nonlinear features of the analyzed samples. In [8], Kumngern et al. propose the implementation of a fifth-order low pass filter for electrocardiogram (ECG) acquisition purposes. 

In [9], Chen et al. propose a novel gas flowmeter based on a microcontroller and a time-to-digital converter aimed at solving common gas flowmeter drawbacks such as pressure loss, reduced range, and contact measurement. In [10], Song et al. proposed the implementation of a nonuniform multiphase time to digital converter, by means of a Cyclone 10 FPGA as a working platform, achieving a resolution of 8.8 ps and showing insensitivity to temperature variations. Another time-to-digital converter implementation is shown by Shin et al. in [11], with the feature to be reconfigurable and the aim to be used in electrical impedance spectroscopy. 

Zamora et al. propose in [12] a complementary metal oxide semiconductor (CMOS)-based analog front-end transceiver for ultra-sonic imaging. The piezoelectric ultrasonic transducer was fabricated on top of the interface substrate. In [13], Li et al. tackle some of the limitations that affect typical CMOS sensors such as linearity degradation caused by in-pixel circuitry and propose the introduction of a pre-distortion circuit based on a nonlinear ramp generator to replace linear ones commonly adopted. 

In [14], Zhang et al. propose a modeling of the temperature hysteresis of inertial navigation systems used in aerial navigation and a novel compensation methodology. Marszalek and Duda, in [15], present the design and performances of a simultaneous and multifrequency impedance measurement system used with four inductive loop sensors for detecting vehicles’ magnetic profile. Finally, in [16], Pettinato et al. propose a current reference circuit based on the LT199x amplifier, which has increased accuracy and linearity with respect to commonly adopted op-amp-based solutions. 

Guest Editors would like to take this opportunity to thank all the authors for having submitted their papers to this special issue and also want to thank all the reviewers for having dedicated their time and helping to improve the quality of the submitted papers.
